# Five-year follow-up of participants in a randomised controlled trial showing benefits from exercise for breast cancer survivors during adjuvant treatment. Are there lasting effects?

**DOI:** 10.1007/s11764-012-0233-y

**Published:** 2012-07-27

**Authors:** Nanette Mutrie, Anna Campbell, Sarah Barry, Kate Hefferon, Alex McConnachie, Diana Ritchie, Sian Tovey

**Affiliations:** 1 School of Psychological Sciences and Health, University of Strathclyde, 76 Southbrae Drive, Glasgow, G13 1PP UK; 2 Institute of Sport and Exercise, University of Dundee, Dundee, UK; 3 Robertson Centre for Biostatistics, University of Glasgow, Glasgow, UK; 4 School of Psychology, University of East London, London, UK; 5 Beatson West of Scotland Cancer Centre, 1052 Great Western Road, Glasgow, G12 0YN UK; 6 Crosshouse Hospital, Kilmarnock, KA2 0BE UK; 7 Present Address: Institute of Sport, Physical Education and Health Sciences, University of Edinburgh, Holyrood Road, Edinburgh, EH8 8AQ UK

**Keywords:** Breast cancer survivors, Physical activity, Five-year follow-up, Quality of life

## Abstract

**Purpose:**

In an earlier randomised controlled trial, we showed that early stage breast cancer patients who received a supervised exercise programme, with discussion of behaviour change techniques, had psychological and functional benefits 6 months after the intervention. The purpose of this study was to determine if benefits observed at 6 months persisted 18 and 60 months later.

**Methods:**

Women who were in the original trial were contacted at 18 and 60 months after intervention. Original measures were repeated.

**Results:**

Of the 148 women from the original study who agreed to be contacted again, 114 attended for follow-up at 18 months and 87 at 60 months. Women in the original intervention group reported more leisure time physical activity and more positive moods at 60 months than women in the original control group. Irrespective of original group allocation, women who were more active consistently reported lower levels of depression and increased quality of life compared to those who were less active.

**Conclusions:**

We have shown that there are lasting benefits to an exercise intervention delivered during treatment to breast cancer survivors. Regular activity should be encouraged for women with early stage breast cancer as this can have lasting implications for physical and psychological functioning.

## Introduction and background

Worldwide, approximately 1.38 million women are diagnosed with breast cancer each year [1]. In developed countries, 80 % of women with breast cancer will survive at least 60 months due to early detection techniques and effective anti-cancer treatments. In the UK, there are currently around 550,000 breast cancer survivors [2].

Breast cancer treatments can cause chronic side effects such as oestrogen deprivation symptoms, athralgias, fatigue, lymphoedema, peripheral neuropathy, reduced bone health, upper extremity functional impairments and overall functional decline [3]. A considerable number of breast cancer survivors experience some of these side effects although there is currently no accurate quantitative data on the incidence of these symptoms. The evidence that exercise is effective in treating many of these chronic or late appearing side effects is compelling: a recent systematic review and meta-analysis supported the use of exercise to prevent or treat fatigue and lymphoedema and to improve functional status and upper body range of movement [4]. In addition, prospective observational studies suggest that around 3 h of aerobic activity per week can significantly reduce the risk of cancer recurrence and breast cancer mortality [5]. There is now a need for randomised controlled trials (RCTs) to examine the long-term effects of exercise interventions for improving outcomes such as quality of life, symptom management and ultimately cancer recurrence and mortality. To date, the longest follow-up with cancer survivors after an exercise intervention is 2 years [6], with most RCTs only following participants up to 6 months post intervention [7]. The aim of this study was to follow-up participants from a RCT during adjuvant treatment 18 and 60 months after the exercise intervention.

The original study’s aim was to determine if there were functional and psychological benefits of a 12-week supervised group exercise programme during treatment for early stage breast cancer, including a 6-month follow-up. The study was designed as a pragmatic randomised controlled prospective open trial and was set in three oncology clinics in Scotland for recruitment and in community facilities for the exercise intervention. The participants were 203 women with breast cancer with 177 completing the 6-month follow-up. The intervention incorporated a variety of safe cardiovascular, muscular strength and flexibility exercises and group discussion of exercise behaviour change techniques, in addition to usual care. The control group received usual care until the 6-month follow-up when they had a one to one discussion about how to incorporate physical activity into their lifestyle. Details of the methods and results are reported in more detail elsewhere [8]. The main outcome measures were: Quality of Life (FACT) questionnaire, Beck Depression Inventory (BDI), positive and negative affect scale (PANAS), body mass index (BMI), 7-day recall of physical activity from the Scottish Physical Activity Questionnaire-2 (SPAQ), 12-min walk test and assessment of shoulder mobility. The results showed significant intervention effects at 12 weeks and 6 months follow-up for metres walked in 12 min, minutes of moderate intensity activity reported in a week, shoulder mobility, breast cancer specific subscale of quality of life and for positive mood. It was concluded that a supervised group exercise programme provided functional and psychological benefit after a 12-week intervention and six months later.

## The aim of the this follow-up study was

1. To determine if intervention effects continued after the 6-month follow-up
2. To determine if women who had higher levels of activity after diagnosis and treatment had a different functional or psychological profile than women who had lower levels of activity and to elicit views from the women concerning their experience of physical activity post intervention

However, in this paper, we will not report on the qualitative analysis of the women’s views. The methods and results for that qualitative aspect will be reported elsewhere.

## Method

### Participants

All women who had participated in the original study and who had agreed to being contacted again (*n* = 148) were contacted at 18 months after the intervention and invited to participate in the follow-up study. Sixty months after the intervention, all women (regardless of 18-month participation) were contacted again and invited to participate in a further follow-up.

### Procedures

Each woman’s General Practitioner (GP) was contacted to ensure it was appropriate to write to the participant. Eligible participants were sent a letter inviting them to take part. Women who agreed to take part were then contacted by telephone to arrange for reassessment at the local sports facility where the original assessments had been carried out. All procedures and outcome measures were identical to the original study. Each appointment lasted approximately 2 h. All procedures were approved by the local NHS research ethics committee and informed consent obtained.

### Data analysis

In the original study, the participants were seen at the beginning and end of the exercise intervention period (i.e. baseline and 3 months) and at the 6-month follow-up (i.e. 6 months from the end of the intervention period). In this study, we add 18 and 60-month follow-up time points.

At each time point, the participants self-reported the total number of minutes of leisure time activity undertaken in the previous week, using a validated questionnaire (SPAQ). At each time point, they were classified as ‘more’ or ‘less’ active if they were above or below, respectively, the sample median leisure time activity for their age group.

Baseline demographics were summarised and compared for those who had and had not dropped out of the study at each follow-up time point.

The effect of the intervention on change from baseline in each outcome was modelled over time using a linear mixed effects model with a random intercept for subject, adjusting for study site, therapy received at baseline and age. The mean difference in change from baseline between the intervention groups at each time point was estimated with a 95 % confidence interval (CI) and *p* value. The model implicitly accounts for missing data by considering the individual trends over time as well as the observed group means at each time point to give a more accurate estimate (than the observed group mean alone) of the population group means over time. For example, if women with lower scores at earlier time points tend to be more likely to drop out later, then the estimated mean at later time points will be adjusted downwards slightly to account for this.

The differences in the outcomes at each time point between the more and less active group were estimated using similar models, additionally adjusting for intervention group. Note that it was not possible to consider change from baseline for this comparison because women were not necessarily in the same activity groups at different time points and so the outcomes were the actual scores rather than change from baseline.

## Results

### Demographics

**Fig. 1:**
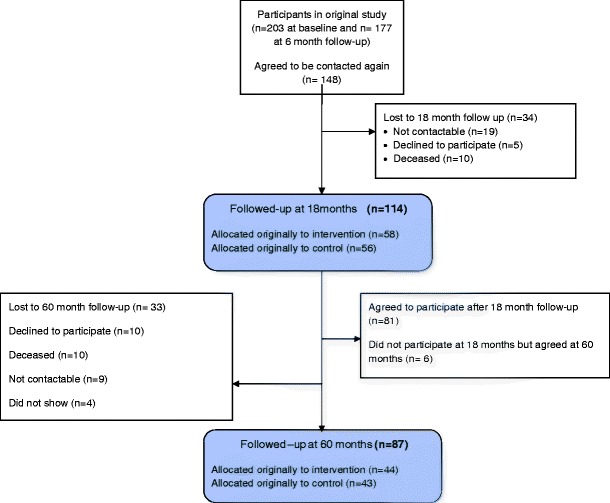
Participant flow through follow-up study

One hundred and fourteen women attended follow-up at 18 months and 87 women attended at 60 months. The flow of participants through this follow-up study is shown in Fig. 1 (see reference 8 for the flow diagram for the original study). Similar numbers of women in each study treatment group participated at 60 months. Baseline demographic characteristics of those that took part in the study at the 18 and 60-month follow-up versus those that did not are shown in Table 1. Those who participated in the follow-up at 60 months were, at baseline, 3 years older and 5 kg lighter on average and were faster walkers (i.e. probably fitter); and may have been slightly less depressed and with less negative mood than those who did not participate at 60 months. Women in work prior to diagnosis and those that were less deprived were more likely to participate than those who were housewives or more deprived. There were no differences in the proportions of control and exercise group women that responded at either 18 or 60 months.

**Table 1:** Demographics at baseline for women that took part in the follow-up study (responders) and women that did not take part in the follow-up study (non-responders) at each subsequent time point: summary statistics and *p* values for differences between responders and non-responders (Wilcoxon/Fisher’s test)

		18 months			5 years		
		Responder		*p*	Responder		*p*
		No	Yes		No	Yes	
Age (years)	*n*	87	114	<0.01	114	87	0.03
	Mean (SD)	49.0 (9.2)	53.5 (9.3)		50.3 (9.5)	53.2 (9.3)	
Baseline weight (kg)	*n*	85	114	<0.01	112	87	<0.01
	Mean (SD)	74.2 (15.5)	68.3 (13.3)		73.2 (15.2)	67.8 (13.2)	
Height (cm)	*n*	87	112	0.20	114	85	0.14
	Mean (SD)	161.0 (6.3)	159.9 (6.0)		160.9 (6.3)	159.7 (5.9)	
BDI score	*n*	85	112	0.06	112	85	0.06
	Mean (SD)	13.3 (7.2)	11.4 (7.1)		13.1 (7.5)	11.1 (6.6)	
PANAS positive	*n*	87	112	0.10	113	86	0.13
	Mean (SD)	26.5 (8.4)	28.9 (9.0)		26.8 (8.6)	29.2 (8.9)	
PANAS negative	*n*	87	112	0.05	113	86	0.09
	Mean (SD)	19.5 (7.9)	17.2 (6.8)		19.1 (7.8)	17.0 (6.5)	
12-min walk (m)	*n*	85	114	0.18	112	87	0.03
	Mean (SD)	973.4 (220.8)	996.0 (224.8)		958.1 (242.4)	1,022.7 (190.0)	
SPAQ leisure time activity (min)	*n*	85	110	0.32	110	85	0.71
	Mean (SD)	367.0 (330.3)	365.1 (267.9)		375.1 (323.2)	354.1 (257.7)	
SRM total score	*n*	87	114	0.80	114	87	0.98
	Mean (SD)	30.7 (5.8)	30.8 (5.3)		30.6 (5.7)	30.9 (5.4)	
BMI (kg/m^2^)	*n*	85	112	0.01	112	85	0.02
	Mean (SD)	28.6 (6.0)	26.8 (5.3)		28.3 (5.9)	26.6 (5.3)	
Exercise group	Control	46/102 (45.1)	56/102 (54.9)	0.67	59/102 (57.8)	43/102 (42.2)	0.78
	Exercise	41/99 (41.4)	58/99 (58.6)		55/99 (55.6)	44/99 (44.4)	
Study centre	GRI	16/33 (48.5)	17/33 (51.5)	0.55	21/33 (63.6)	12/33 (36.4)	0.57
	BOC	62/151 (41.1)	89/151 (58.9)		85/151 (56.3)	66/151 (43.7)	
	Other	9/17 (52.9)	8/17 (47.1)		8/17 (47.1)	9/17 (52.9)	
Therapy	Chemotherapy	7/15 (46.7)	8/15 (53.3)	0.52	7/15 (46.7)	8/15 (53.3)	0.67
	Radiotherapy	21/57 (36.8)	36/57 (63.2)		32/57 (56.1)	25/57 (43.9)	
	Combination	59/129 (45.7)	70/129 (54.3)		75/129 (58.1)	54/129 (41.9)	
Surgery type	Mast only	31/57 (54.4)	26/57 ( 45.6)	0.18	38/57 (66.7)	19/57 ( 33.3)	0.20
	Lump only	48/116 (41.4)	68/116 ( 58.6)		65/116 (56.0)	51/116 ( 44.0)	
	Lump and Mast	1/2 (50.0)	1/2 ( 50.0)		1/2 (50.0)	1/2 ( 50.0)	
	Lump and Recon	0/1 ( 0.0)	1/1 (100.0)		0/1 ( 0.0)	1/1 (100.0)	
	Mast and Recon	6/22 (27.3)	16/22 ( 72.7)		9/22 (40.9)	13/22 ( 59.1)	
	Other	1/2 (50.0)	1/2 ( 50.0)		1/2 (50.0)	1/2 ( 50.0)	
Tamoxifen used	No	57/117 (48.7)	60/117 (51.3)	0.08	72/117 (61.5)	45/117 (38.5)	0.15
	Yes	30/83 (36.1)	53/83 (63.9)		42/83 (50.6)	41/83 (49.4)	
Highest education level	School	40/92 (43.5)	52/92 (56.5)	1.00	54/92 (58.7)	38/92 (41.3)	0.77
	Other	43/99 (43.4)	56/99 (56.6)		55/99 (55.6)	44/99 (44.4)	
Employment status (prior to diagnosis)	FT/PT	10/29 (34.5)	19/29 (65.5)	0.01	11/29 (37.9)	18/29 (62.1)	0.02
	Sick	52/111 (46.8)	59/111 (53.2)		66/111 (59.5)	45/111 (40.5)	
	Housewife	17/26 (65.4)	9/26 (34.6)		20/26 (76.9)	6/26 (23.1)	
	Retired	8/35 (22.9)	27/35 (77.1)		17/35 (48.6)	18/35 (51.4)	
Occupation (prior to diagnosis)	Professional	17/48 (35.4)	31/48 (64.6)	0.72	21/48 (43.8)	27/48 (56.2)	0.39
	Managerial	14/35 (40.0)	21/35 (60.0)		18/35 (51.4)	17/35 (48.6)	
	Clerical	25/55 (45.5)	30/55 (54.5)		32/55 (58.2)	23/55 (41.8)	
	Manual	15/33 (45.5)	18/33 (54.5)		20/33 (60.6)	13/33 (39.4)	
Carstairs’ deprivation	1–2	17/58 (29.3)	41/58 (70.7)	0.04	23/58 (39.7)	35/58 (60.3)	0.01
	3–5	42/86 (48.8)	44/86 (51.2)		53/86 (61.6)	33/86 (38.4)	
	6–7	26/53 (49.1)	27/53 (50.9)		35/53 (66.0)	18/53 (34.0)	
Periods	No	70/169 (41.4)	99/169 (58.6)	0.36	95/169 (56.2)	74/169 (43.8)	0.74
	Irregular	8/17 (47.1)	9/17 (52.9)		9/17 (52.9)	8/17 (47.1)	
	Regular	9/15 (60.0)	6/15 (40.0)		10/15 (66.7)	5/15 (33.3)	
Hysterectomy	No	80/179 (44.7)	99/179 (55.3)	0.36	101/179 (56.4)	78/179 (43.6)	1.00
	Yes	7/22 (31.8)	15/22 (68.2)		13/22 (59.1)	9/22 (40.9)	
HRT	Never	58/124 (46.8)	66/124 (53.2)	0.31	73/124 (58.9)	51/124 (41.1)	0.69
	Former	24/67 (35.8)	43/67 (64.2)		35/67 (52.2)	32/67 (47.8)	
	Current	5/10 (50.0)	5/10 (50.0)		6/10 (60.0)	4/10 (40.0)	

### Effects of the intervention

**Fig. 2:**
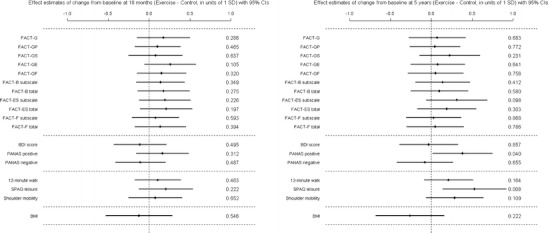
Exercise treatment effect estimates for all outcomes at 18 and 60 months, adjusted for original study site, therapy received at baseline and baseline age, with 95 % confidence intervals (CIs) and corresponding *p* values at the right hand side

To determine if there were any lasting effects of the intervention, comparisons were made between the original treatment and control groups. We have already reported the intervention benefits 6 months after intervention. Table 2 shows descriptive statistics of the outcome data at 18 and 60 months with corresponding treatment effect estimates. There were significant differences between the intervention and control groups at 60 months for SPAQ leisure time activity over the previous week and PANAS positive mood score with the intervention group reporting higher activity and more positive mood. Even for outcomes for which there was no significant difference at 60 months, the intervention group was consistently observed to do better than the control group throughout the entire 5-year follow-up period. The treatment effect estimates at 18 and 60 months are also displayed in units of 1 standard deviation in Fig. 2 for all outcomes measured and are of similar magnitude at both time points. At 5 years, the intervention group achieved on average around 200 min of activity each week more than the control group (see Table 2 for related data). In general, our analyses suggested that 5 years subsequent to taking part in such an exercise intervention similar patients would be likely to achieve on average 50 to 350 min of extra physical activity per week than patients treated as usual. This is a substantial difference which could lead to considerable health benefit.

**Table 2:** Main outcomes: summary statistics at 18 and 60 months for the control and exercise intervention groups and model effect estimates^a^ of treatment effect differences for change from baseline

			Summaries at each time point			Effect estimate (Exercise − Control)	
			Baseline	18 months	5 years	18M − Baseline	5Y − Baseline
FACT-G	All	*n*	201	114	87		
		Mean	74.4	80.7	87.1		
		(SD)	(14.3)	(14.6)	(11.1)		
	Control	*n*	102	56	43	2.2	0.9
		Mean	72.7	79.6	85.7	(−1.8, 6.2)	(−3.4, 5.2)
		(SD)	(15.6)	(14.7)	(11.4)	0.286	0.683
	Exercise	*n*	99	58	44		
		Mean	76.1	81.7	88.5		
		(SD)	(12.6)	(14.6)	(10.7)		
BDI score	All	*n*	197	114	87		
		Mean	12.2	9.3	7.0	−0.8	−0.2
		(SD)	(7.2)	(7.7)	(6.7)	(−3.1, 1.5)	(−2.8, 2.4)
	Control	*n*	98	56	43	0.495	0.857
		Mean	12.9	9.7	7.5		
		(SD)	(7.5)	(7.7)	(6.7)		
	Exercise	*n*	99	58	44		
		Mean	11.5	8.9	6.6		
		(SD)	(6.9)	(7.8)	(6.7)		
PANAS positive	All	*n*	199	114	87		
		Mean	27.8	31.0	34.2		
		(SD)	(8.8)	(9.8)	(8.3)		
	Control	*n*	100	56	43	1.5	3.4
		Mean	28.0	30.6	33.1	(−1.4, 4.3)	(0.2, 6.7)
		(SD)	(9.2)	(10.1)	(8.7)	0.312	0.040
	Exercise	*n*	99	58	44		
		Mean	27.7	31.3	35.2		
		(SD)	(8.4)	(9.6)	(7.8)		
PANAS negative	All	*n*	199	114	87	−0.8	−0.5
		Mean	18.2	16.7	15.4	(−2.9, 1.4)	(−2.9, 1.8)
		(SD)	(7.4)	(7.5)	(5.3)	0.487	0.655
	Control	*n*	100	56	43		
		Mean	19.1	17.4	16.3		
		(SD)	(7.7)	(8.1)	(5.6)		
	Exercise	*n*	99	58	44		
		Mean	17.3	16.0	14.5		
		(SD)	(6.9)	(6.9)	(5.0)		
12-min walk	All	*n*	199	95	83		
		Mean	986	1,085	1,065		
		(SD)	(223)	(192)	(158)		
	Control	*n*	100	47	40	20	40
		Mean	975	1,066	1,031	(−33, 74)	(−16, 97)
		(SD)	(235)	(169)	(163)	0.463	0.164
	Exercise	*n*	99	48	43		
		Mean	997	1,104	1,096		
		(SD)	(211)	(213)	(147)		
SPAQ leisure time activity (min)	All	*n*	195	111	84	79	204
		Mean	366	533	557	(−48, 206)	(54, 354)
		(SD)	(296)	(355)	(321)	0.222	0.008
	Control	*n*	99	55	41		
		Mean	365	500	462		
		(SD)	(288)	(334)	(263)		
	Exercise	*n*	96	56	43		
		Mean	367	565	648		
		(SD)	(306)	(373)	(347)		
SRM total score	All	*n*	201	110	86		
		Mean	30.7	32.4	32.8		
		(SD)	(5.5)	(5.3)	(5.1)		
	Control	*n*	102	53	43	0.3	1.2
		Mean	30.3	31.7	31.8	(−1.1, 1.7)	(−0.3, 2.7)
		(SD)	(5.7)	(5.6)	(5.9)	0.652	0.109
	Exercise	*n*	99	57	43		
		Mean	31.2	33.1	33.8		
		(SD)	(5.4)	(5.0)	(3.9)		
BMI	All	*n*	197	111	85	−0.3	−0.6
		Mean	27.6	27.5	27.3	(−1.2, 0.7)	(−1.6, 0.4)
		(SD)	(5.7)	(5.1)	(5.4)	0.546	0.222
	Control	*n*	100	56	43		
		Mean	27.7	28.0	28.0		
		(SD)	(6.1)	(6.4)	(6.7)		
	Exercise	*n*	97	55	42		
		Mean	27.4	26.9	26.7		
		(SD)	(5.3)	(3.3)	(3.7)		

*18M* 18 months follow-up, *5Y* 60 months follow-up

^a^Effects estimates are displayed as the mean estimate, 95 % confidence interval and *p* value. Model adjusted for study site, baseline therapy and age

Adjusting for other baseline variables (such as deprivation category, occupation prior to diagnosis, hysterectomy status, work status) had negligible effect on the group differences for any of the outcomes, despite some of these baseline variables showing strong relationships with the outcomes and/or differing between women that were followed up at 18 and 60 months and those that were not.

### Associations with levels of self-reported activity

To determine if there were differences between those women who self-reported themselves as being ‘more’ active and those who self-reported that they were ‘less’ active at each follow-up point, comparisons were made between these two categories of women, adjusting for original treatment group (as well as baseline study site, therapy and age).

**Fig. 3:**
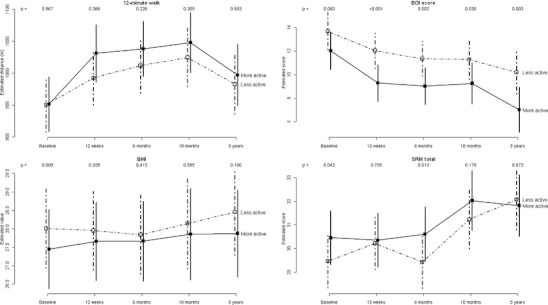
Model-estimated mean 12-min walk distance, Beck depression inventory score, BMI and shoulder range of motion score over time for the more and less active groups, adjusted for original study site, therapy received at baseline and baseline age, with *p* values for tests of differences between the groups at each time point

**Fig. 4:**
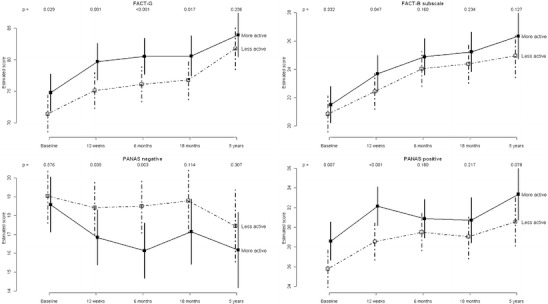
Model-estimated mean FACT-G, FACT-B subscale, PANAS positive and PANAS negative scores over time for the more and less active groups, adjusted for original study site, therapy received at baseline and baseline age, with *p* values for tests of differences between the groups at each time point

The model-estimated trends for the main outcomes over all time points, with confidence intervals, are given in Figs. 3 and 4. Figure 3 illustrates that the more active group was observed to walk a slightly longer distance in 12 min at every follow-up time point, though the differences were not significant.

The BDI score was marginally significantly different between the groups at baseline and decreased for both groups over time. A larger decrease in depression levels for the group identifying as active was associated with significant differences at all follow-up points. BMI scores were on average slightly lower for the active group throughout the study, though the difference was statistically significant only at baseline and 12 weeks. There were statistically significant differences between the activity groups for total shoulder range of motion at baseline and 6 months follow-up only, and the observed difference was not consistent over time.

Figure 4 shows similar increases in FACT-G average scores (and therefore quality of life improvements) for both activity groups over time. In general, by 60 months follow-up there were no statistically significant differences in any of the quality of life scales, despite the consistency of the observed difference over time.

**Fig. 5:**
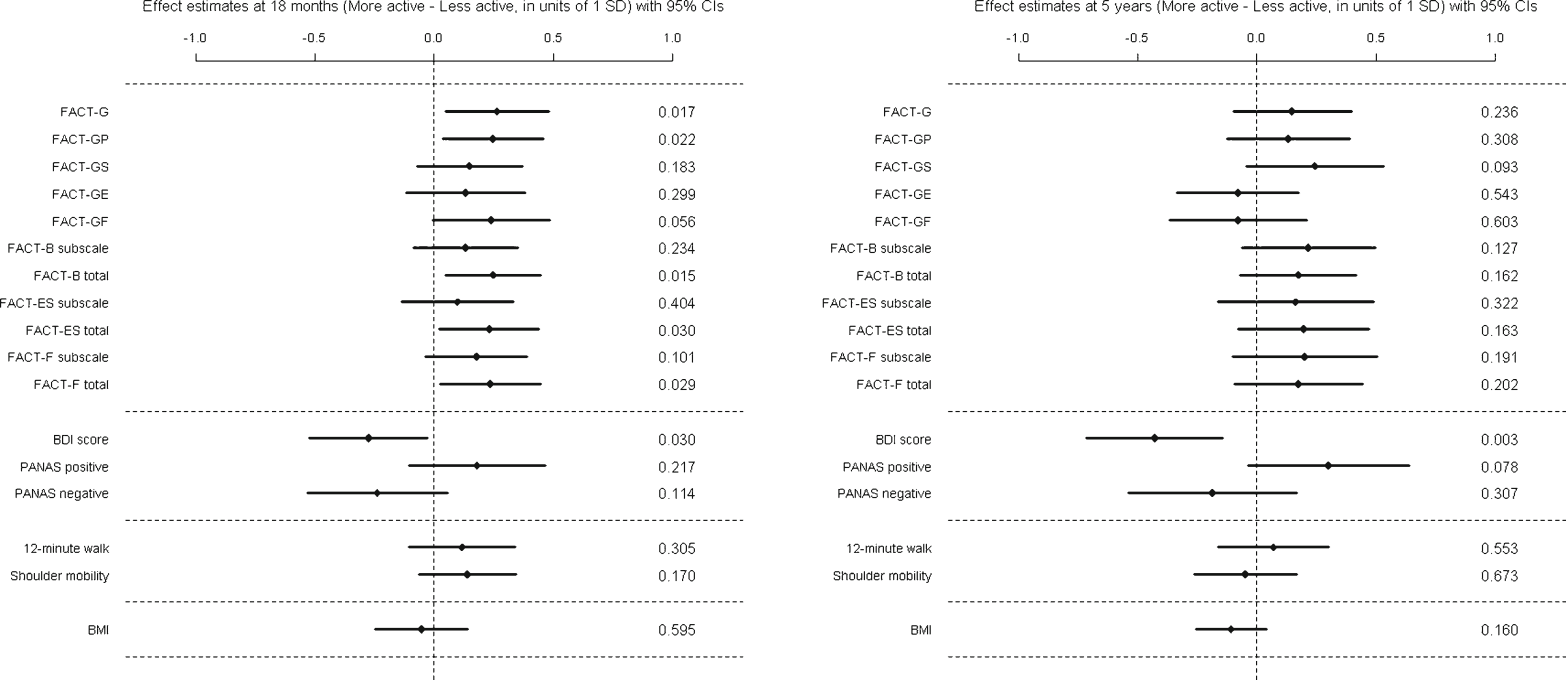
Physical activity effect estimates for all outcomes at 18 and 60 months, adjusted for original study site, therapy received at baseline and baseline age, with 95 % confidence intervals (CIs) and corresponding *p* values at the right hand side

PANAS negative was significantly lower in the more active group at the end of the original study period and this persisted out to 6 months follow-up, despite there being no difference at baseline. This difference was not, however, statistically significant at 18 or 60 months, though the observed difference remained similar over time. Similarly, the more active group had significantly higher PANAS positive at baseline and at the end of the original study period and though the magnitude of this difference was similar at 60 months, it was marginally non-significant (0.078). The physical activity effects estimates, in units of 1 standard deviation, are displayed in Fig. 5 for all of the outcomes at 18 and 60 months.

## Discussion

This is the first study to examine the long-term effects of an exercise intervention in a RCT with cancer survivors. The number of women lost to follow-up at 18 (44 %) and 60 months (58 %) is higher than that observed in a 2-year follow-up of a 6-month rehabilitation programme to reduce lymphoedema after breast cancer surgery (27 %) [6] but similar to that found in a longitudinal study of older breast cancer survivors in a 6-year follow-up (50 %) [9]. Five years after taking part in the study, women who were assigned to the original intervention group, who had received the opportunity to attend a 12-week programme of supervised group exercise and group discussion of behaviour change issues, self-reported more leisure time activity and more positive mood than those women originally assigned to the control group condition. This is very encouraging as our trial was designed to promote long-term exercise behaviour change. Both the intervention and control groups self-reported high levels of physical activity at 5 years exceeding current public health recommendations of achieving 150 min of moderate activity in a week. Thus even the control group has benefitted from being involved in this project 5 years after diagnosis. However, as the data from Table 2 shows, the intervention group reported around 200 min of activity more than the control group at 5 years, and although there is also high variability in these data, this is a statistically significant difference. The possibility of over reporting physical activity because of the self-report nature of the data must be acknowledged but the difference between the two groups in terms of physical activity is substantial. This suggests that the experience of attending the group exercise sessions had influenced the ability to sustain physical activity at a high level for the intervention group. This increase in physical activity could have important additional physical and mental well-being effects such as improved mood which we have observed, and reduced risk of recurrence of breast cancer, improved bone health and biomarker levels (e.g. insulin pathway and inflammation) which were not measured in the original study.

An important element of the exercise programme was the group discussions that happened at the end of each class. Each week, for 6 weeks, a specific theme was covered in group discussion after the exercise (for example, the health benefits of exercise, enhancing self-efficacy and setting goals) and supported with specifically constructed materials. These themes were guided by a model of behaviour change and were designed to promote independent exercise after the intervention [10]. The six week block was repeated on a rolling basis, allowing all participants to hear the same themes. At the end of the 12-week intervention, the women were helped to construct an individual exercise programme. The control group received a personal consultation after the 6-month follow-up about how to increase physical activity levels. After the final data collection, women from both groups who expressed an interest in a local exercise referral scheme were given information on how to attend. The results show that the original intervention had a long lasting effect on helping the intervention group maintain a more physically active life. The difference in physical activity level that we see at 60 months between intervention and control group can be attributed to the experience of the class and group discussion of behaviour change challenges and solutions.

In a study of cancer survivors diagnosed more than 5 years ago [11], over 53 % reported difficulties in crouching, standing for 2 h, carrying 10 pounds and walking quarter of a mile—compared to 21 % of a matched sample with no cancer history. This demonstrates the importance of helping cancer survivors maintain basic levels of physical performance for simple activities of daily living. Positive mood is an indication of psychological well-being and may also be linked to increased activity levels. Williams et al. [12] found that an acute positive affective response to a single bout of moderate intensity exercise predicted physical activity participation levels 6 and 12 months later. This is consistent with other follow-up studies [7] and recent meta-analysis [4] which suggest positive effects of exercise on psychosocial parameters. Overall the pattern of results suggests a range of benefits of participating in the supervised exercise programme providing that the programme includes discussion of behaviour change challenges and solutions. The results therefore support the implementation of exercise opportunities into cancer rehabilitation in the same way that exercise is now a mainstream component of cardiac rehabilitation.

Irrespective of original group allocation, those who self-reported as engaging in higher levels physical activity recorded benefits on many of the quality of life and mood variables in comparison to those who self-reported that they were less active. This suggests that being active, regardless of original group allocation to intervention or control conditions, was associated with quality of life and mood benefits. A 13-year follow-up of 374 women diagnosed with breast cancer at a young age (<40) showed that the women whose exercise activity increased following diagnosis scored significantly higher (*p* = 0.005) on the SF-36 physical health quality of life scale [13]. Likewise a prospective study investigating physical activity and quality of life in 545 breast cancer survivors showed that greater physical activity levels 3 years post diagnosis were related to less fatigue and better physical functioning [14]. In general, statistically significant differences are more apparent at 18-month follow-up than at 60 months, though it is important to note that the number of women responding at 60 months was lower and the magnitude of the effect for several outcomes is similar to the corresponding 18-month effect.

### Strengths and limitations

This is first study to follow an intervention group for 60 months after an exercise intervention for women with early stage breast cancer and our response rate is similar to other studies of this length. A limitation is that there were some differences in baseline demographics and outcome scores between those that did and did not return for follow-up and a reasonably high rate of dropout at 60 months. However, we used statistical modelling methods that appropriately accounted for such missing data to give reliable estimates of the population group means and corresponding differences over time, and we adjusted the models for baseline demographics.

Physical activity measures in this study were self-reported and future studies should attempt objective monitoring of physical activity patterns including sedentary time.

## Conclusions

Some of the benefits of a supervised exercise programme that incorporated discussion of behaviour change techniques, which were reported 6 months following the original intervention, have remained 60 months after the original study ended. These include higher levels of self-reported leisure time activity and more positive mood for the intervention group in comparison to the original control group. Categorising the women by self-reported activity status, rather than by original allocation to intervention and control conditions, also shows benefits over time in terms of lower levels of depression and higher levels of mood and quality of life for those who report being more active. Cancer survivors should be encouraged to engage in regular physical activity and to work towards achieving the public health recommendations for sufficient physical activity during and after treatment for early stage breast cancer [15, 16]. Services to support regular physical activity might include supervised exercise sessions in early stages, similar to that provided for cardiac rehabilitation, and encouragement to make use of local physical activity opportunities.
